# The use of alpha 1 thymosin as an immunomodulator of the response against SARS-Cov2

**DOI:** 10.1186/s12979-023-00351-x

**Published:** 2023-07-05

**Authors:** M. S. Espinar-Buitrago, L. Tarancon-Diez, E. Vazquez-Alejo, E. Magro-Lopez, M. Genebat, F. Romero-Candau, M. Leal, M. A. Muñoz-Fernandez

**Affiliations:** 1 Immunology Section, Laboratorio Inmuno-Biología Molecular (LIBM), Hospital General Universitario Gregorio Marañón (HGUGM), Instituto de Investigación Sanitaria Gregorio Marañón (IiSGM), 28009 Madrid, Spain; 2 Centro de Investigación Biomédica en Red Bioingeniería, Biomateriales y Nanotecnología (CIBER-BBN), Madrid, Spain; 3 Department of Internal Medicine, Hospital Fátima, 41012 Sevilla, Spain; 4 Department of Internal Medicine, Hospital Viamed Santa Ángela de la Cruz, 41014 Seville, Spain; 5 Home Residencia de la Santa Caridad, 41001 Seville, Spain

## Abstract

**Background:**

Since the beginning of SARS-CoV2 pandemic, the mortality rate among elderly patients (60–90 years) has been around 50%, so age has been a determining factor of a worse COVID-19 prognosis. Associated with age, the thymic function involution and depletion plays an important role, that could be related to a dysregulated and ineffective innate and adaptive immune response against SARS-CoV2. Our study aims to further in vitro effect of human Thymosin-alpha-1 (α1Thy) treatment on the immune system in population groups with different thymic function levels in the scenario of SARS-CoV2 infection.

**Results:**

Activation markers such as CD40, CD80 and TIM-3 were upregulated in α1Thy presence, especially in plasmacytoid dendritic cells (pDCs) and, with increased TNFα production was observed compared to untreated condition. Co-cultures of CD4 + and CD8 + T cells with DCs treated with α1Thy in response to SARS-CoV2 peptides showed a decrease in the cytokine production compared to the condition without α1Thy pre-treated. A decrease in CD40L activation co-receptor expression in CD8 + LTs was also observed, as well as an increase in PD1 in CD4 + TLs expression in both age groups. In fact, there are no age-related differences in the immunomodulatory effect of the hormone, and it seems that effector memory and terminally differentiated memory T lymphocyte subsets were the most actively influenced by the immunomodulatory α1Thy effect. Finally, the polyfunctionality measured in SARS-CoV2 Specific-T cells response was maintained in α1Thy presence in total and memory subpopulations CD4 + and CD8 + T-cells, despite decreased proinflammatory cytokines production.

**Conclusion:**

The hormone α1Thy could reduce, through the modulation of DCs, the amount of proinflammatory cytokines produced by T cells. Moreover, α1Thy improve lymphocyte functionality and could become a beneficial therapeutic alternative as an adjuvant in SARS-CoV2 treatment either in the acute phase after infection or reinfection. In addition, the effect on the T immune response means that α1Thy can be incorporated into the vaccination regimen, especially in the most immunologically vulnerable individuals such as the elderly.

**Subjects:**

Thymosin alpha 1, Dendritic cells, SARS-CoV2-specific T cells response, Immunomodulation

**Supplementary Information:**

The online version contains supplementary material available at 10.1186/s12979-023-00351-x.

## Background

Since it began in 2019, the global pandemic caused by SARS-CoV2 has been a critical threat to global health, straining health systems worldwide. Target cells infection by SARS-CoV2 resulting in the activation of host innate and adaptive immune cells, triggering the proinflammatory cytokines and chemokines production from epithelial and effector immune cells, leading to immune system dysfunction [1]. Dysregulations of the immune system by COVID-19 disease, such as lymphopenia and cytokine storm, have been associated with disease severity, suggesting a pivotal role of the host response in pathogenesis [2]. Although the application of different vaccines has improved the situation created by COVID-19, a treatment against the disease that modulates the immune system, improving the main effects especially in severe patients, such as elderly people, has not really been established.

It is well known that advanced age is a mortality predictor and severe clinical outcomes in SARS-CoV2 infection. In this regard, one of the age-dependent factors is the loss of thymic function and thus the individual's immune response to fight diseases [3]. Innate and adaptive immunosenescence correlates with a reduced ability to generate antigen-specific and vaccine responses that could results in an increased incidence of infections, neoplastic and autoimmune diseases [4]. Age-associated thymic involution involves a decrease in tissue mass and thymic naïve T cells production. These age-related changes in peripheral T cells are thought to contribute significantly to the features of immunosenescence, suggesting that altered thymic activity is a key factor in the decline of immune function in the elderly [5, 6]. In fact, our group has recently hypothesised that there is an association between age-related loss of thymic function and the severity of and response to SARS-CoV2 infection [7, 8].

Regarding human Thymosin-alpha-1 (α1Thy), has emerged as a new therapeutic approach in SARS-CoV2 infection [9, 10]. This hormone is secreted by thymic epithelial cells and is widely distributed throughout secondary lymphoid organs modifying immune function and playing an important role in immune cell activation and regulation as an immune modulator [11, 12]. Clinical treatment of α1Thy has been tested in a variety of settings with an excellent safety profile for use in diseases with impaired or reduced immune response like sepsis [13], chemotherapy-induced immunosuppression [14, 15], treatment of immunological disease such as cystic fibrosis [16], and even infection with viruses such as human immunodeficiency virus (HIV-1) [17], hepatitis B virus (HBV) [18] and hepatitis C virus (HCV) [19, 20], also, α1Thy has been used as an adjuvant in vaccinations, showing an increase in antibody response in vaccination against some viral pathogens such as influenza virus [21, 22].

This led to the inclusion of α1Thy as a treatment for SARS-CoV2 infection during the pandemic in China. Although its use also generated some doubts because its effects on severe COVID19 patients’ immune system was not clear [23, 24], another favourable clinical approaches using α1Thy in this context has recently been reported, showing clinical benefit and immunological recovery [25]. It appears that this hormone has demonstrated its effect in promoting thymus immune cell production, as well as restoring lymphocytopenia and acute cellular depletion, during α1Thy administration in patients with COVID-19 [26].

This study aims to determine, in vitro*,* the immunomodulatory effects of α1Thy on the innate and adaptive immune system in the scenario of SARS-CoV2 infection.

## Results

### α1Thy increases activation and maturation of DCs and decreases cellular depletion

**Fig. 1:**
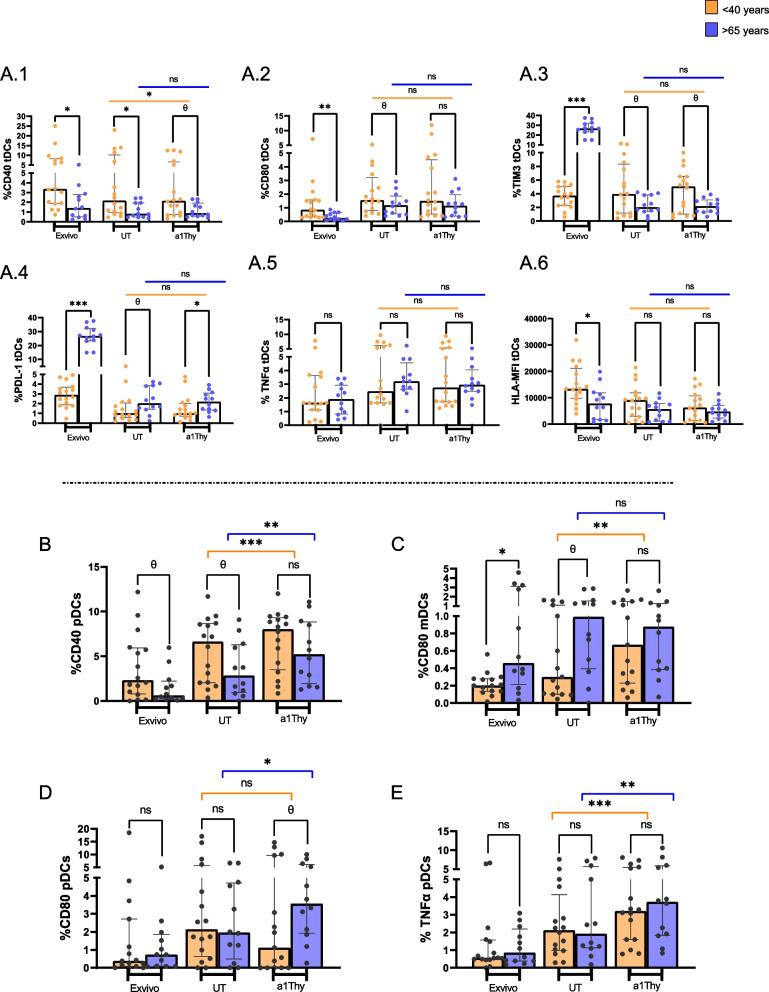
Immunophenotyping of DCs. DCs phenotype and activation, maturation and exhaustion markers and intracellular production of TNFα on DCs. Bar and scatter dots graphs represent the expression of each biomarker in total DCs: CD40, CD80, TIM-3, PDL-1 (**A.1-A.4**); TNFα intracellular production (**A.5**); HLA-MFI (**A.6**). The bar graphs represent marker expression: CD40 on plasmacytoid dendritic cells (pDCs) (**B**); CD80 on myeloid dendritic cells (mDCs) (**C**); CD80 on pDCs (**D**) and intracellular production of TNFα on pDCs (**E**). The medians with the interquartile ranges are shown. Ex-vivo: Ex-vivo condition; UT: Untreated condition; α1Thy: α1Thy treated condition. Each dot represents an individual. Orange dots represent < 40 years (*n* = 18) and > 65 years (*n* = 16) are highlighted with blue dots. Wilcoxon test was used comparing condition in the same group and U-Mann Whitney test was used comparing condition between different group (ns: not statistically significative, θ: *p* > 0.05 and < 0.1 *p < 0.05; **p < 0.01; ***p < 0.001)

The results regarding DCs activation showed a CD40 upregulation in < 40-years group but no significant differences in > 65-years group comparing α1Thy treatment condition with untreated condition, also no differences in CD80 and in TIM-3 expression in both groups were observed (Fig. 1).

Regarding PDL-1 expression, intracellular TNFα production and HLA-DR mean fluorescence intensity in total DCs did not differ between the treated and untreated conditions in both groups (Fig. 1A1-6). However, when we studied mDCs and pDCs subpopulations we found a significant increase at CD40 expression in pDCs from both age groups. CD80 levels experienced a significant increase in mDCs for < 40-years group and in pDCs in > 65-years group**.** No differences were observed for TIM-3 or PDL-1, nor in the HLA-DR mean fluorescence intensity in subpopulations of either study group. Finally, when we studied the intracellular TNFα production, although no differences were observed in any study condition for mDCs, there was a significant increase in the production of this cytokine in pDCs in the treatment with α1Thy in both age groups (Fig. 1B-E). To confirm the existence of differences in markers’ expression and TNFα production in total DCs comparing the two age groups, a fold change was performed to analyse the difference between conditions with and without α1Thy treatment. The results showed that there are significant differences in the expression of CD40, TIM-3 as well as PDL-1 in total DCs. For DCs subpopulations, significant differences were observed in CD40 expression on pDCs and in CD80 expression on mDCs, maintaining a trend in pDCs for CD80, as well as PDL-1 expression on mDCs (Additional File 1).

### α1thy decreases cytokine production by CD4 + T and CD8 + T cells in response to SARS-CoV2 peptides

**Fig. 2:**
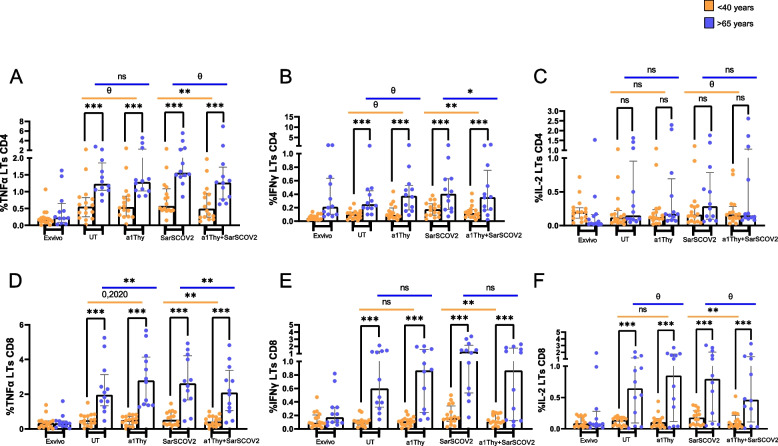
Immunophenotyping and cytokine production on total CD4 + and CD8 T cells. Intracellular production of TNFα, IFNγ, and IL-2 in T cells was evaluated from co-culture of CD4 + or CD8 + TLs in response to SARS-CoV2 peptides with autologous DCs previously treated with α1Thy. Bar and Scatter plots graphs represent percentage of cytokine production: in total CD4 TLs TNFα, IFNγ, and IL-2 (**A-C**); in total CD8 TLs: TNFα, IFNγ, and IL-2 (**D-F**). The medians with the interquartile ranges are shown. Ex-vivo: Ex-vivo condition; UT: Untreated condition; α1Thy: α1Thy treated condition; SARS-CoV2: SARS-CoV2 peptides stimulation condition; α1Thy + SARS-CoV2: α1Thy treated and SARS-CoV2 peptides stimulation condition. Each dot represents an individual. Orange dots represent < 40 years (*n* = 18) and > 65 years (*n* = 16) are highlighted with blue dots. Wilcoxon test was used comparing condition in the same group and U-Mann Whitney test was used comparing condition between different group (ns: not statistically significative, θ: *p* > 0.05 and < 0.1 *p < 0.05; **p < 0.01; ***p < 0.001)

To determine the nature of immune responses induced by DCs previously treated with α1Thy, we co-cultured DCs with autologous CD4 + or CD8 + TLs and determined the production of cytokines by T lymphocytes stimulated or not with a pool of SARS-CoV2 peptides (Fig. 2).

The results showed that there are significant differences in the levels of cytokine production in the different study conditions when comparing the < 40-years group and the > 65-years group. In elderly group, elevated TNFα, IFNγ and IL-2 production was observed in total CD4 + and CD8 + T cells in all the condition we studied. When comparing the conditions with SARS-CoV2 peptides stimulation with or without α1Thy pre-treatment, a significant decrease in TNFα and IFN-γ production in total CD4 + T cells was observed in the < 40 age group, with the same trend for IL-2 production (*p* = 0.0907). On the other hand, the > 65 age group experienced a trend of decreased TNFα production in CD4 + T cells (*p* = 0.0992), as well as a significant decrease in IFN-γ production in total CD4 + T cells, with no change in IL-2 production (Fig. 2A-C). Regarding inflammatory cytokine production in CD8 + T cells, the < 40 years’ group experienced a decrease in all three cytokines studied, TNF-α, IFN-γ and IL-2. The > 65 years’ group experienced a significant decrease in TNFα production and a trend towards decreased IL-2 production (*p* = 0.0725), with no change observed for IFN-γ when comparing stimulation conditions with SARS-CoV2 peptide in the presence or absence of α1Thy (Fig. 2D-F).

**Fig. 3:**
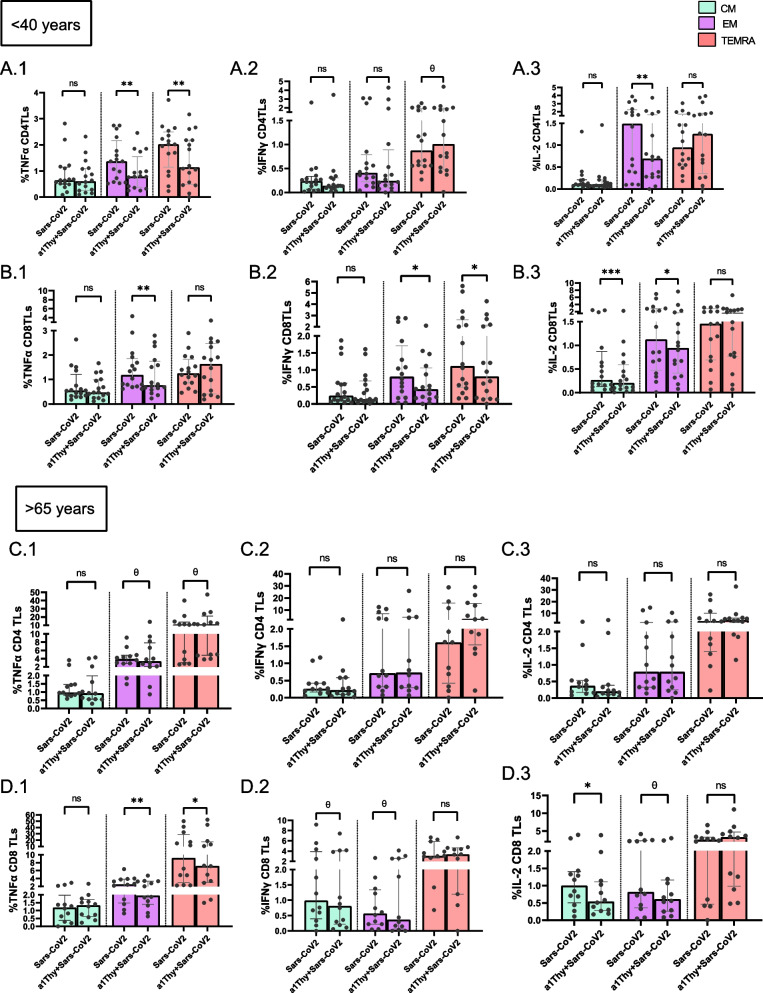
Immunophenotyping and cytokine production on CD4 + and CD8 memory T cells. Intracellular production of TNFα, IFNγ, and IL-2 in T cells was evaluated from co-culture of CD4 + or CD8 + TLs in response to SARS-CoV2 peptides with autologous DCs previously treated with α1Thy. Bar and Scatter plots graphs represent percentage of cytokine production: in memory populations of CD4 + TLs in < 40 years’ group: TNFα, IFNγ, and IL-2 (**A.1-A.3**); in memory populations CD8 + TLs in < 40 years’ group: TNFα, IFNγ, and IL-2 (**B.1-B.3**); in memory populations of CD4 + TLs in > 65 years’ group: TNFα, IFNγ, and IL-2 (**C.1-C.3**); in memory populations CD8 + TLs in > 65 years’ group: TNFα, IFNγ, and IL-2 (**D.1-D.3**). The medians with the interquartile ranges are shown. Ex-vivo: Ex-vivo condition; UT: Untreated condition; α1Thy: α1Thy treated condition; SARS-CoV2: SARS-CoV2 peptides stimulation condition; α1Thy + SARS-CoV2: α1Thy treated and SARS-CoV2 peptides stimulation condition. Each dot represents an individual. Memory populations represented in green central memory T-cells (CM), in violet effector memory T-cells (EM) and in red terminally differentiated effector memory T cells (TEMRA). Wilcoxon test was used comparing condition in the same group and U-Mann Whitney test was used comparing condition between different group (ns: not statistically significative, θ: *p* > 0.05 and < 0.1 *p < 0.05; **p < 0.01; ***p < 0.001)

In addition, analysis of the memory populations was carried out focusing on the comparison between the SARS-CoV2 peptide-stimulated condition without and with α1Thy pre-treatment in CM, EM and TEMRA memory TLs populations (Fig. 3).

For < 40-years group, the results showed a significant decrease of TNF-α production in EM and TEMRA CD4 + TLs populations, as well as a decrease of IL-2 in EM comparing the presence and the absence of α1Thy. However, a tendency to increase IFN-γ production in TEMRA CD4 + T cells is observed (*P* = 0.0734) (Fig. 3A.1- 2A.3). The results also showed a significant decrease of TNF-α production in EM, as well as a decrease of IFN-γ production in EM and TEMRA and a decrease of IL-2 production in CM and EM CD8 + TLs populations (Fig. 3B.1- B.3). Regarding the memory populations of CD4 + TLs in > 65-years group, no changes in cytokine production were observed in the presence or absence of α1Thy treatment, except for a decreasing trend in EM and an increasing trend in TEMRA for TNF-α production (*p* = 0.0978 and *p* = 0.0742, respectively) (Fig. 3C.1-C.3). Although, a decrease in proinflammatory cytokines production is observed in different memory populations of CD8 + T lymphocytes. Thus, we observed a significant decrease for TNF-α production in EM and TEMRA, a trend in CM and EM for IFN-γ production (*p* = 0.09658 and *p* = 0.0803 respectively) and a significant decrease in CM for IL-2 production, which also showed some tendency to decrease in EM (*p* = 0.0883) (Fig. 3D.1-D.3).

To confirm the existence of differences in cytokines production between the two age groups comparing the conditions with and without α1Thy treatment and stimulation with SARS-CoV2 peptides a fold change was performed. The results showed that the treatment works in a similar way in both groups and no differences were observed between them except in TNF-α production in both CD4 + and CD8 + total T cells. However, in memory population, we observed that there were differences between the age groups in TNFα production in TEMRA CD4 + TLs, a tendency in IFN-γ production in EM CD8 + TLs and some tendency in IL-2 production in all memory populations in CD8 + T cells (Additional File 2).

**Fig. 4:**
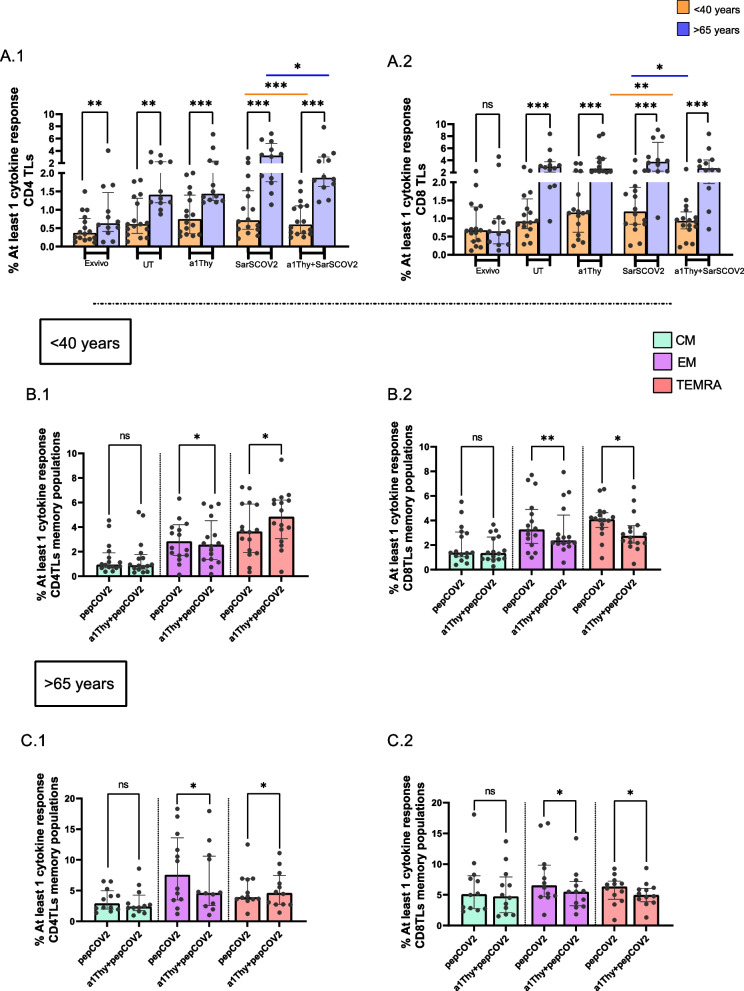
At least 1 cytokine production in total and memory populations of T lymphocytes. Bar graphs represent percentage of at least 1 cytokine production in total CD4 TLs and total CD8 TLs (**A.1** and **A.2**). Bar graphs describe the percentage of at least 1 cytokine production in memory populations includes central memory T-cells (CM), effector memory T-cells (EM) and terminally differentiated effector memory T cells (TEMRA)for CD4 and CD8 T cells in < 40-years group (**B.1** and **B.2**) and for CD4 and CD8 T cells in > 65-years group (**C.1** and **C.2**). The medians with the interquartile ranges are shown. Ex-vivo: Ex-vivo condition; UT: Untreated condition; α1Thy: α1Thy treated condition; SARS-CoV2: SARS-CoV2 peptides stimulation condition; α1Thy + SARS-CoV2: α1Thy treated and SARS-CoV2 peptides stimulation condition. Each dot represents an individual. Orange dots represent < 40 years (*n* = 18) and > 65 years (*n* = 16) are highlighted with blue dots. Memory populations represented in green (CM), in violet (EM) and in rose (TEMRA). Wilcoxon test was used comparing condition in the same group and U-Mann Whitney test was used comparing condition between different group (ns: not statistically significative, θ: *p* > 0.05 and < 0.1 *p < 0.05; **p < 0.01; ***p < 0.001)

In addition, inflammatory cytokines production was also studied as a mathematical summation of the probabilities of producing at least 1 of the studied cytokines (TNF-α, IFN-γ and IL-2) by lymphocytes (Fig. 4).

The results showed a significant decrease of at least one cytokine production in total CD4 + and CD8 + TLs populations in both age groups when we compared the study conditions, stimulated with SARS-CoV2 peptides without and with α1Thy treatment (Fig. 4A.1 and A.2). In addition, we also studied the memory populations of both lymphocyte groups and observed that EM population of CD4 + and CD8 + T lymphocytes showed a significant decrease in the production of at least one cytokine, although the TEMRA population of CD4 + TLs acts by significantly increasing inflammatory cytokine production at < 40-years group and > 65-years group (Fig. 4B.1 and C.1). In contrast, TEMRA population of CD8 + TLs decreases proinflammatory cytokine production in the presence of the hormone in both age groups (Fig. 4B.2 and C.2).

### Activation and depletion of DCs-T-cell interaction regulated by αThy effect

**Fig. 5:**
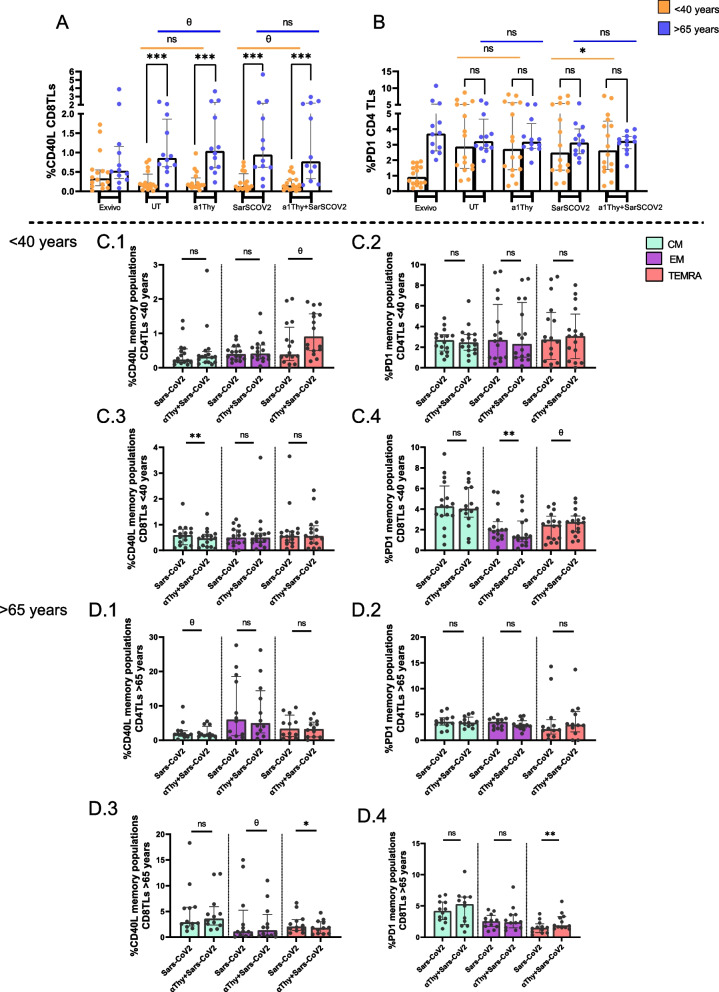
Expression of activation and exhaustion markers in T cells. Co-receptors were evaluated from co-culture of CD4 or CD8 TLs in response to SARS-CoV2 peptides with autologous DCs previously treated with α1Thy. Bar and scatter plots graphs represent the expression of CD40L co-receptor CD8 total T cells (**A**) and PD1 co-receptor in CD4 T cells (**B**). Bar graphs describe the expression of each co-receptor in memory populations: In < 40 years’ group CD40L expression in Central Memory, Effector Memory and Terminally Differentiated Memory (CM, EM and TemRA, respectively) CD4 T-cells (**C.1**); PD1 expression in CM, EM and TemRA CD4 T-cells respectively (**C.2**); CD40L expression in CM, EM and TemRA, respectively in CD8 T-cells (**C.3**); PD1 expression in CM, EM and TemRA, respectively CD8 T-cells (**C.4**). In > 65 years’ group CD40L expression in CM, EM and TemRA, respectively CD4 T-cells (**D.1**); PD1 expression in CM, EM and TemRA CD4 T-cells respectively (**D.2**); CD40L expression in CM, EM and TemRA, respectively in CD8 T-cells (**D.3**); PD1 expression in CM, EM and TemRA, respectively CD8 T-cells (**D.4**) The medians with the interquartile ranges are shown. Ex-vivo: Ex-vivo condition; UT: Untreated condition; α1Thy: α1Thy treated condition; SARS-CoV2: SARS-CoV2 peptides stimulation condition; α1Thy + SARS-CoV2: α1Thy treated and SARS-CoV2 peptides stimulation condition. Each dot represents an individual. Orange dots represent < 40 years (*n* = 18) and > 65 years (*n* = 16) are highlighted with blue dots. Memory populations represented in green (CM), in violet (EM) and in rose (TEMRA). Wilcoxon test was used comparing condition in the same group and U-Mann Whitney test was used comparing condition between different group (ns: no statistically significative, θ: *p* > 0.05 and < 0.1 *p < 0.05; **p < 0.01; ***p < 0.001)

We also wanted to study the interaction between DCs and TLs through different co-receptors that act also like activation markers, CD40 ligand (CD40-L), and depletion programmed cell death factor (PD1) (Fig. 5).

The results showed a decreasing trend in CD40L expression on total CD8 + T cells (*p* = 0.0829) (Fig. 5A) and an increase in PD-1 on CD4 + T cells in < 40-years group (Fig. 5B). However, no others significant differences were observed in this same age group comparing SARS-CoV2 peptides in absence or presence of αThy conditions. In > 65 donors’ group there did not appear to be significant differences for CD40L or PD1 in total T cells, although we observed changes at memory populations. In < 40-years group, the results showed that there was a tendency for CD40L expression to increase in TEMRA (*p* = 0.0587) but no differences in PD1 expression were observed in any of CD4 + T cell memory populations. However, more alterations were observed in CD8 + T cells memory populations showing a significant decrease in CD40L levels in CM population and a significant decrease in PD1 expression in EM (Fig. 5C.1-C.4). In > 65-years group, we observed a tendency to increase CD40L expression in EM (*p* = 0.0649) but any difference in PD1 expression on memory population in CD4 + T cells. Furthermore, in CD8 + TLs memory populations there was a significant decrease in CD40L expression but a tendency to increase PD1 expression in TEMRA (*p* = 0.0611) (Fig. 5D.1-D.4).

**Fig. 6:**
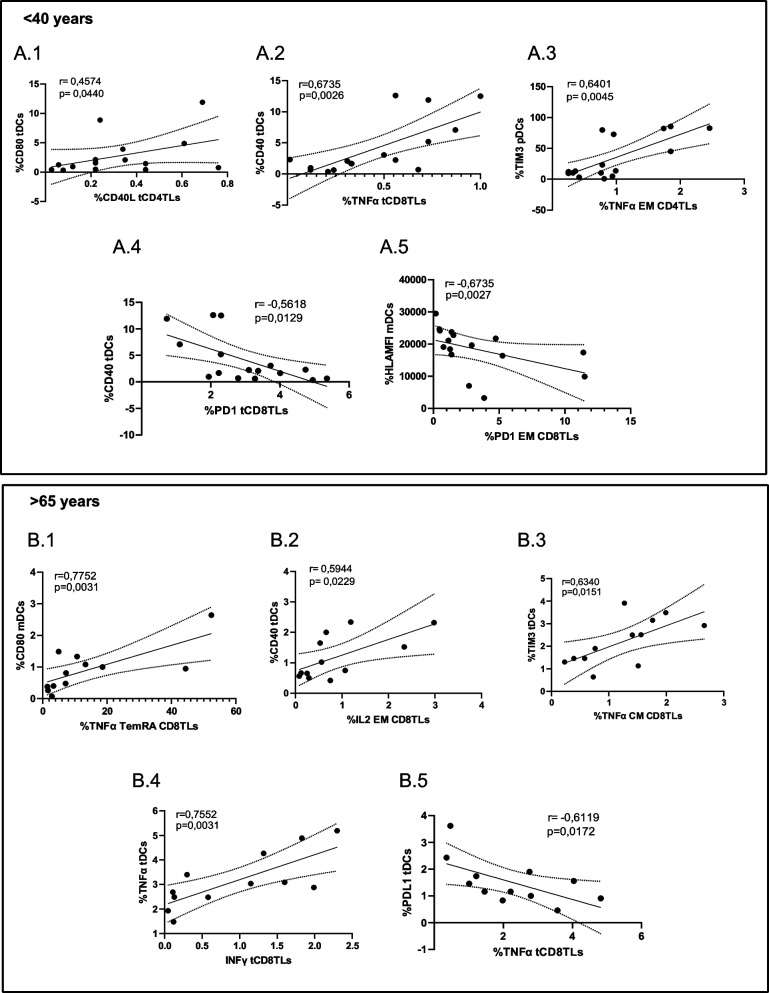
Correlations between markers in DCs and cytokines production, activation and exhaustion of TLs. In < 40 age group, association between CD80 in total DCs and CD40L in total CD4 T cells, respectively (**A.1**); CD40 in total DCs and TNFα production in total CD8 T cells (**A.2**); TIM-3 in pDCs and TNFα production in EM of CD4 TLs (**A.3**); CD40 in total DCs and PD1 in CD8 in TLs (**A.4**) and between HLA fluorescence intensity in mDCs and PD1 in EM of CD8 TLs (**A.5**). In > 65 age group, association between CD80 in mDCs and of TNFα production in TEMRA CD8 TLs (**B.1**); CD40 in total DCs and IL-2 production in EM CD8 TLs (**B.2**); TIM-3 in total DCs and of TNFα production in CM CD8 TLs (**B.3**); TNFα in total DCs and IFNγ production in CD8 TLs (**B.4**) and PDL-1 in total DCs and the TNFα production in CD8 TLs (**B.5**). The spearman rho correlation coefficient was used

In addition, we studied the possible correlations in both age groups for stimulated with SARS-CoV2 peptides before treatment with α1Thy condition (Fig. 6).

Results in < 40-years group showed positive correlations between the activation marker CD80 on total DCs and CD40L ligand on CD4 + T cells (Fig. 6A.1) and between CD40 on total DCs and TNFα intracellular production on total CD8 + T cells (Fig. 6A.2). There was also a positive correlation between the expression of TIM-3 expression in pDCs and TNFα in EM CD4 + T lymphocytes (Fig. 6A.3). Negative correlations were also observed between CD40 expression in total DCs and PD1 expression in CD8 + T lymphocytes (Fig. 6A.4), as well as between HLA fluorescence intensity in mDCs and this co-receptor (Fig. 6A.5). For elderly group, similar correlations described in the young group were observed between CD80, CD40 and even TIM-3 expression in DCs and TNFα and IL-2 production in CD8 + TLs memory subpopulations (Fig. 6B.1-B.3). In addition, other correlations were observed between TNFα produced in DCs and IFNγ production in CD8 + TLs (Fig. 6B.4). Negative correlations were established between PDL-1 in total DCs and TNFα production in CD8 + T lymphocytes (Fig. 6B.5). These correlations show that there was a relationship between DCs treated with α1Thy and lymphocytes stimulated with SARS-CoV2 peptides.

### Lymphocyte functionality is enhanced by the modulatory effect of the hormone α1Thy

**Fig. 7:**
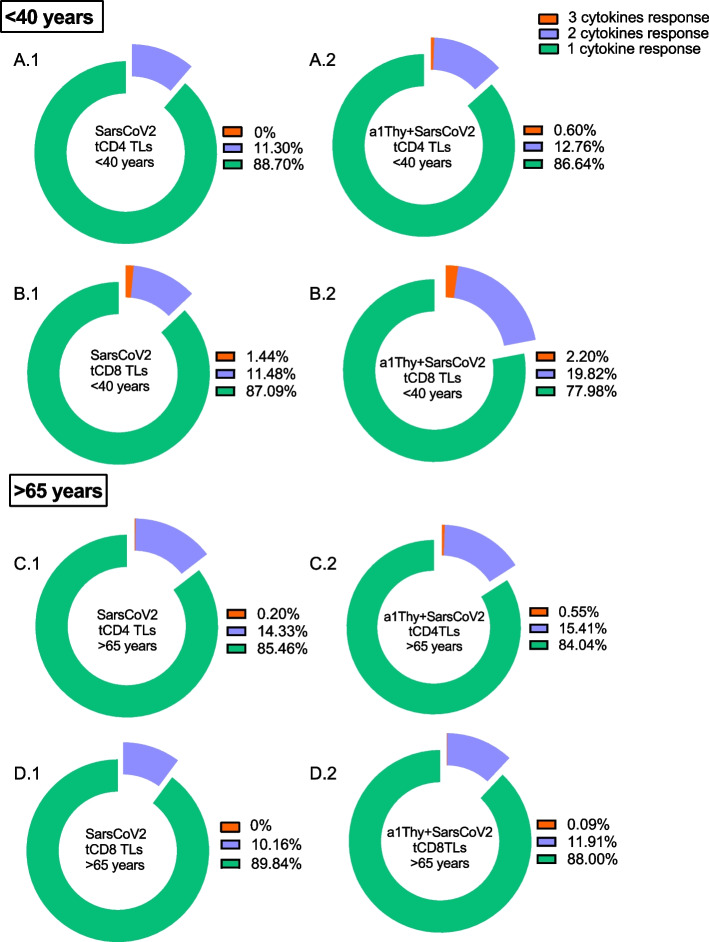
Polyfunctionality in CD4 + and CD8 + T cells. Polyfunctionality patterns of CD4 and CD8 total T cells response to SARS-CoV2 producing one (green), two (blue) or three (orange) functions (combination of TNFα, IFNγ and IL2) among the different condition (with or without α1Thy treatment) for the two study groups: < 40 years (CD4 TLs: **A.1-A.2**; CD8 TLs: **B.1-B.2**) and > 65 years (CD4 TLs: **C.1-C.2**; CD8 TLs: **D.1-D.2**)

We studied the quality of the lymphocyte response by studying polyfunctionality understood as the capacity of LTs to produce more than one cytokine simultaneously. The results were obtained by mathematical analysis of the three cytokines (TNFα, IFNγ and IL-2) production possible combinations in total CD4 + and CD8 + LTs (Fig. 7).

The results showed that polyfunctionality was maintained in CD4 + T lymphocytes in < 40-years group (Fig. 7A.1 and A.2). However, in CD8 + LTs was showed that there was an increase in triple function and double function when comparing the SARS-CoV2 peptide-stimulated condition with the α1Thy-treated and SARS-CoV2 peptide-stimulated condition (Fig. 7B.1 and B.2). Moreover, in CD4 + T and CD8 + T lymphocytes in > 65 years (Fig. 7C.1-D.2) no showed major differences, but a slight increase in the double and triple lymphocyte function was observed.

Analysing the memory populations polyfunctionality, we observed that CM, EM and TEMRA CD4 + T cells memory populations suffered an increase in double and triple function in the young group (Additional File 3A.1-A.6), being more pronounced in the TEMRA population. For CD8 + T cells, no changes in polyfunctionality were observed in triple function memory populations for < 40-years group (Additional File 3B.1-B.6), with the exception of the EM population where a slight decrease in triple function was observed and an increase in double function. In > 65-years group, minor changes were observed in CD4 + (Additional File 3C.1-C.6) and CD8 + T (Additional File 3D.1-D.6) cells.

## Discussion

Thymic involution and immunosenescence has been factors of worse prognosis in SARS-CoV2 disease due to a deregulated and ineffective innate and adaptive immune response against the virus [27]. Our results show that in vitro treatment with α1Thy upregulate CD40, CD80, TIM-3 expression and increase TNFα production in DCs. Also, decrease TNFα, IFNγ and IL-2 production in T cells co-cultured with DCs and a decrease CD40L co-receptor expression as well as increase PD1 expression in both age groups. So, α1Thy attempts to reverse cellular depletion and modulates cytokine production, establishing a balance between inflammation and enhanced immune response. In fact, there are no age-related differences in the immunomodulatory effect of the hormone, and it seems that effector memory and terminally differentiated memory T lymphocyte subsets were the most actively influenced by the immunomodulatory α1Thy effect. In addition, in a1Thy presence there is an improvement in lymphocyte functionality by decreasing the amount of pro-inflammatory cytokines but maintaining the quality of the response.

Age-associated loss of thymic function leads to lower activation of DCs due to an impaired immune response [28]. According with our results, exogenous introduction of the hormone may enhance antigen-presenting function equalising the response between younger and older populations. It appears that α1Thy is increasing the expression of activation markers in DCs, especially in the pDC subpopulation the major are the major ones involved in antiviral response [29], as well as increasing TNFα production as a mechanism to enhance the immune response as antigen-presenting cells (APCs) [30]. Previous studies have showed how α1Thy hormone is a very effective promoter of antiviral immune responses by interacting with signalling of different Toll like receptors (TLR) present in immune system cells such as DCs, macrophages and natural killers (NKs) cells becoming a mediator of innate and adaptive immunity and activating intracellular signalling pathways as NF-κB, p38 MAPK, and MyD88-dependent [31, 32]. It seems that the hormone can activate the TLR9/MyD88 signalling pathway through the induction of TNFα production which activates CD4 + and CD8 + T cells initiating pathogen elimination (antiviral effect) [33].

SARS COV2 antigenic recognition by TLRs could generate the massive and polyfunctional release of proinflammatory cytokines triggering respiratory distress and multiorgan failure as a clinical outcome [34]. Nowadays and thanks to the inclusion of vaccines, clinical symptoms are milder, however, there is still a percentage of patients who develop complications, especially in the case of elderly patients [35]. Due to the immunostimulatory effect of the α1Thy hormone, it could be understood that may exert an agonist effect worsening the patient's condition; however, some studies show how α1Thy interferes in the MYD88/TRIF pathway avoiding the cytokine dysregulation that occurs when TLRs are stimulated by SARS-COV2 antigens appears and results in cytokine storm and T-cell exhaustion [33, 36]. In that sense, our results show a decrease in the production of TNFα, IFNγ and IL-2 when we studied the specific T response against SARS-COV2 after α1Thy pre-treatment, especially in CD8 + T lymphocytes in both age groups, equalizing the immunomodulatory effect observed in older volunteers with younger donors. More in depth, the distribution of lymphocyte memory populations is different between CD4 + and CD8 + T lymphocytes among younger and elderly groups, such that there is a higher percentage of terminally differentiated memory population (TEMRA) in elderly study group, in detriment of the "naive" population, which in part could explain the lower immune response to new antigens in elderly [37, 38]. However, our studies show that the effector memory (EM) population has the greatest influence on the T response in the presence of hormone in both the < 40 and > 65 years’ groups, although in the > 65-years group the TEMRA population also influences the specific T response [39, 40]. In addition, most marked effect is generated on CD8 T lymphocytes, so it seems that α1Thy tries to modulate the cytotoxic response by controlling the proinflammatory cytokine production levels when are excessively elevated. We observed that TNFα is the proinflammatory cytokine most diminished by the effect of the hormone, followed by IL-2 in both study groups in EM and TEMRA CD4 + TLs populations. However, IFNγ plays a dual role, being even increased in the memory populations of CD4 + TLs and decreased in the case of CD8 + TLs. Moreover, the intensity of the specific T response will depend to a large extent on the immune characteristics of each person. Donors who show less response after stimulation, independently of age, with SARS-CoV2 peptides show less decrease in proinflammatory cytokines production and, in some cases, even a slight increase in the presence of the hormone, especially in the CD4 + TLs. Therefore, the effect of the α1Thy hormone is mainly to decrease the excess production of proinflammatory cytokines generated by SARS-CoV2 stimulation, especially in case of CD8 + cytotoxic T cells, although some CD4 + T populations act in the opposite way in order to maintain the immune response.

Previous observations demonstrate how the hormone α1Thy specifically modulate in human lymphocytes the expression of several genes encoding cytokines, chemokines, and molecules of innate and adaptive immunity [41]. This modulation effect on the production of proinflammatory cytokines may be due to the interaction established between the co-receptors between DCs and TLs in the presence of the hormone [42]. When we analyse these co-receptors, we observe correlations between different markers and cytokine production, so that we could associate the immunomodulatory effect to the PDL1-PD1 interaction, as a control point for cytokine production and above all the CD40-CD40L interaction, which generates an activation signalling cascade promoting the production of cytokines. In this sense, significant differences observed in the elderly group, in an attempt to control the overproduction of proinflammatory cytokines, especially TNFα, inside a group with a higher degree of cellular senescence and a persistent low-grade inflammation called “*inflammaging*” with increased production of baseline cytokines [43]. Furthermore, the decrease in cytokine production with the absence of variation in polyfunctionality demonstrate that α1Thy is able to maintain the quality of the lymphocyte response, and this is through the immune regulation exerts on T lymphocytes.

The effect we observe in the presence of α1Thy is more pronounced in case of youngest, although the trends are maintained in both age groups. These differences could be due to the immune system deterioration that occurs with age, as well as the presence of comorbidities in > 65 years’ donors may have, but above all in this study to the presence of higher endogenous α1Thy values in younger donors [44]. Some studies carried out with older patients show that despite the atrophy of the thymus suffered by this population and the senescence phenomenon, when an α1Thy regimen is established, it is capable of stimulating thymic function, which implies an improvement in the immune response during SARS-CoV2 infection, even in the most severe cases, including sustained use, that could be capable of preventing infection, being a clear adjuvant in elderly people [45, 46]. In this study, donors involved had overcome SARS-CoV2 infection at the time of extraction but were able to respond to a new viral stimulation, as could be the case in a reinfection despite the presence of vaccination.

This study has some limitations such the size of the study groups. We did not have control groups (< 40 years and > 65 years) that despite vaccination had not undergone SARS-CoV2 infection, which means that we do not have data on the effect of α1Thy in healthy donors without SARS-CoV2 infection. However, despite these limitations, it seems that α1Thy hormone may exerts a beneficial effect as an adjuvant treatment, both in the infection and in the generation of a better specific T response.

## Conclusions

In summary, in this in vitro study the α1Thy hormone appears to have a potential immunomodulatory role, and could improves the maturation and activation of DCs as APCs. In addition, it may decrease the production of SARS-CoV2 specific inflammatory cytokines while enhances the differentiation and activation of T cells TLs, allowing a balance between decreasing inflammation and correct antiviral activity. It also appears to improve the quality of the lymphocyte response, enable a correct immune response to be restored and is a key factor in elderly patients. However, further studies are needed to verify whether immunocompromised patients could benefit from treatment with α1Thy, either during reinfections with the different viral variants or even, as has already been demonstrated for influenza virus in elderly people, as an adjuvant in vaccination procedures.

## Material and methods

### Study participants

Donors from Hospital General Universitario Gregorio Marañón (Madrid, Spain) and Hospital Residencia de la Caridad (Sevilla, Spain) were recruited following these inclusion criteria: 1) Asymptomatic subjects diagnosed during the last 18 months with SARS-CoV2 by RT-PCR + or SARS-CoV2 anti-IgM/IgG + test, younger than 40 years-old (*n* = 18); 2) Asymptomatic subjects diagnosed during the last 18 months with SARS-CoV2 by positive RT-PCR + or SARS-CoV2 anti-IgM/IgG + test older than 65 years-old (*n* = 16).

The study was approved by the Ethics Committee of Hospital General Universitario Gregorio Marañón (HGUGM). Written informed consent was obtained from all donors before inclusion in the study.

### Cell and plasma separation

Participants' peripheral blood mononuclear cells (PBMCs) were separated from peripheral blood samples (30 ml) collected from participants (*n* = 32) in ethylenediaminetetraacetic acid (EDTA) tubes using Ficoll (Ficoll- PaqueTM PLUS) by density gradient centrifugation on the day of blood collection and used immediately for the isolation of DCs, CD4 + and CD8 + T lymphocytes (TLs).

### Cell isolation and stimulation

Fresh DCs and CD4 + and CD8 + LTs were isolated from PBMCs by negative immunoselection using the EasySep Human Pan-DC Pre-Enrichment Kit, EasySep Human CD4 + T Cell Isolation Kit and EasySep Human CD8 + T Cell Isolation Kit, respectively, following the manufacturer's instructions (Stem Cell). After isolation, cells were suspended in 10% RPMI medium (RPMI 1640 supplemented with 10% heat-inactivated fetal bovine serum (FBS), 100 U/ml penicillin G, 100 µl/ml streptomycin sulphate and 1% l-glutamine). A total of 50,000 DCs were cultured overnight in RPMI 10% without any stimulus (hereafter referred to as "unstimulated condition", US) or with thymosin-alpha-1 (α1Thy) (MyBioSource) at 50 ng/ml in 96-well round-bottom plates at 37 °C/5% CO2. As a positive control, DCs were stimulated with 1 µM CpG-A (ODN 2216; InvivoGen).

For the in vitro co-culture system, after DCs stimulation, autologous CD4 + or CD8 + TLs were added to the α1Thy-stimulated or unstimulated DCs conditions at a DC:TL ratio of 50.000:150.000 for 6 h with 1 µg/ml anti-CD28/CD49d, 0.7 µg/ml monensin (BD Biosciences) and 10 µg/ml brefeldin A (Biolegend) at 37 °C/5% CO2 in the presence or absence of 6 nmoles/peptide of a PepTivator® SARS-CoV-2 Select—premium grade (Miltenyi).

### Flow cytometry

For flow cytometry of DCs cells, both immediately isolated DCs (ex vivo condition) and α1Thy-treated/non-treated conditions were washed with 3% PBS-BSA and surface stained for 30 min using the following: LIVE/DEAD Fixable Aqua Blue Dead Cell Stain (Life Technologies) anti-HLA-DR PerCP (clone L243) (BioLegend), anti-CD80 BV421 (clone L307. 4) (BD Biosciences), anti-PDL1 PECF594 (clone MIH1) (BD Biosciences), anti-CD11c AF700 (clone B-ly6) (BD Biosciences), anti-TIM-3 PE (clone 7D3) (BD Biosciences), anti-CD40 Pe-Cy7 (clone 5C3) (BD Biosciences), anti-CD123 AF488 (clone) (R&D Systems). DCs were then washed, permeabilized and fixed with the Cytofix/Cytoperm kit (BD Biosciences) and stained intracellularly for 30 min with anti-TNFα APC (clone Mab11) (BioLegend). Isotype controls for CD80, PDL-1, TIM-3 and CD80 were included in each experiment. Viable DCs cells were characterised by HLA-DR expression and myeloid dendritic cells (mDCs) and plasmacytoid dendritic cells (pDCs) were defined as HLA-DR + CD11c + CD123- and HLD-DR + CD11c-CD123 + respectively (Additional File 4. A1-7).

For immunophenotyping of TLs and intracellular cytokine staining, ex vivo CD4 + or CD8 + TLs and co-culture conditions stimulated and unstimulated by SARS-CoV2 peptides in vitro were washed with 3% PBS-FBS and surface stained for 30 min using the following LIVE/DEAD Fixable Aqua Blue Dead Cell Stain (Life Technologies), anti-CD45RA ECD (clone 2H4) (Beckman Coulter), anti-CD27 APC-Cy7 (clone M-T271) (BioLegend), anti-CD40-L PE-Cy7 (clone 24–31) (BioLegend) and anti-PD1 FITC (clone EH12. 2H7) (BioLegend). Then cells were washed, permeabilized and fixed with the Cytofix/Cytoperm kit and stained intracellularly for 30 min with anti-TNFα APC (clone Mab11) (BioLegend), anti-IL-2 PE (MQ1-17H12) (BioLegend), anti-IFNγ BV421 (clone B27) (BioLegend) and anti-CD3 PerCP-Cy5.5 (clone SK7) (BD Biosciences). For each co-culture condition, CD4 + or CD8 + TLs were identified as viable CD3 + TLs and the distribution of the memory subset was defined according to CD45RA and CD27 expression as naive (Naïve; CD45RA + CD27 +), central memory (CM; CD45RA-CD27 +), effector memory (EM; CD45RA-CD27) and terminally differentiated memory (TEMRA; CD45RA + CD27-) (Additional File 4. B1-6). Isotype controls for CD40-L and PD1 were included in each experiment. Cells were analysed with a Gallios cytometer (Beckman Coulter) and data were analysed with FlowJo 8.7.7 (TreeStar).

### Statistical analysis

The Statistical Package for the Social Sciences software (SPSS 20.0, Chicago, IL, USA) was used for the statistical analysis. Graphs were generated using GraphPad Prism 9.0 (GraphPad Software, Inc., San Diego, CA, USA). Continuous variables were expressed as medians and interquartile ranges (IQR). Differences between non matched-pairs values were determined using two-tailed Mann Whitney U-test. Wilcoxon matched-pairs signed-rank test was conducted to compare α1Thy treatment effect for each group. The Spearman’s rank test was used to analyse correlations between variables. *P*-values < 0.05 were considered statistically significant.

## Supplementary Information

**Additional file 1. **Fold change in DCs immunophenotyping. Scatter plot and fold change represents the difference between α1Thy treated condition and untreated condition in the marker expression on total DCS: CD40; CD80; TIM-3; PDL-1(A.1-D.1); intracellular production of TNFα(E.1) and intensity of fluorescence of HLADR(F:1). On mDCs: CD40; CD80; TIM-3; PDL-1(A.2-D.2); intracellular production of TNFα (E.2) and intensity of fluorescence of HLADR(F:2). On pDCs: CD40; CD80; TIM-3; PDL-1(A.3-D.3); intracellular production of TNFα(E.3) and intensity of fluorescence of HLADR(F.3). The medians with the interquartile ranges are shown. Ex-vivo: Ex-vivo condition; UT: Untreated condition; α1Thy: α1Thy treated condition. Each dot represents an individual. Orange dots represent < 40 years (*n* = 18) and > 65 years (*n* = 16) are highlighted with blue dots. U-Mann Whitney test was used comparing condition between different group (ns: no statistically significative, θ: *p* > 0.05 and < 0.1 *p < 0.05; **p < 0.01; ***p < 0.001).

**Additional file 2. **Fold change on total and memory CD4 + and CD8 + T lymphocytes. Scatter plot and fold change represents the difference between the SARS-CoV2 peptide-stimulated and α1Thy-pretreated condition and the SARS-CoV2 peptide-stimulated condition of cytokine production in total CD4 TLs: TNFα(A.1), IFNγ(B.1) and IL-2(C.1) and total CD8 TLs: TNFα(D.1), IFNγ(E.1) and IL-2(F.1); in memory CD4 TLs (CM,EM and TEMRA): TNFα(A.2-A.4); IFNγ(B.2-B.4); IL-2(C.2-C.4) and in memory CD8 TLs (CM,EM and TEMRA): TNFα(D.2-D.4); IFNγ(E.2-E.4); IL-2(F.2-F.4): The medians with the interquartile ranges are shown. Ex-vivo: Ex-vivo condition; UT: Untreated condition; α1Thy: α1Thy treated condition; SARS-CoV2: SARS-CoV2 peptides stimulation condition; α1Thy + SARS-CoV2: α1Thy treated and SARS-CoV2 peptides stimulation condition. Each dot represents an individual. Orange dots represent < 40 years (*n* = 18) and > 65 years (*n* = 16) are highlighted with blue dots. Memory populations represented in green (CM), in violet (EM) and in rose (TEMRA). Wilcoxon test was used comparing condition in the same group and U-Mann Whitney test was used comparing condition between different group (ns: not statistically significative, θ: *p* > 0.05 and < 0.1 *p < 0.05; **p < 0.01; ***p < 0.001).

**Additional file 3. **Polyfunctionality in memory CD4 + and CD8 + T cells. Polyfunctionality patterns of CD4 + and CD8 + memory T cells response to SARS-CoV2 producing one (green), two (blue) or three (orange) functions (combination of TNFα, IFNγ and IL-2) among the different condition (with or without α1Thy treatment) for < 40 years’ group in memory CD4 TLs (CM, EM and TEMRA) (A.1-A-A.6) and in memory CD8 TLs (CM, EM and TEMRA) (B.1-B.6) and for > 65 years in memory CD4 TLs (CM, EM and TEMRA) (C.1-A-C.6) and in memory CD8 TLs (CM, EM and TEMRA) (D.1-D.6).

**Additional file 4. **DCs and T-cells gating strategy from a donor. DCs gating strategy and pseudocolor plot representation of surface markers expression CD40, CD80, PDL1, TIM-3, TNFα production and HLADR intensity fluorescence histogram (A.1-A.7). T-cell gating strategy and pseudocolor plot representation of intracellular cytokine production TNFα, IFNγ, IL-2 and surface marker expression CD40L and PD1 (B.1-B.6). UT: untreated condition (red); α1Thy: α1Thy treated condition (blue); SARS-CoV2: SARS-CoV2 peptides stimulated condition (red); α1Thy + SARCoV2: α1Thy treated and SARS-CoV2 peptides stimulated condition (blue).

## Data Availability

All data generated or analysed during this study are included in this published article [and its supplementary information files].

## Abbreviations

α1Thy: Thymosin alpha 1
APCs: Antigen-Presenting Cells
CM: Central Memory T cells
DCs: Dendritic Cells
EM: Effector Memory T cells
mDCs: Myeloid Dendritic Cells
NKs: Natural Killers
PBMCs: Peripheral Blood Mononuclear Cells
pDCs: Plasmacytoid Dendritic Cells
TEMRA: Terminally Differentiated Memory T cells
TLs: T Lymphocytes
TLRs: Toll Like Receptors
