# Supplementary figures and images for: The use of alpha 1 thymosin as an immunomodulator of the response against SARS-Cov2
Source: Immun Ageing. 2023 Jul 5;20:32. doi: 10.1186/s12979-023-00351-x (PMC10320944; doi:10.1186/s12979-023-00351-x)

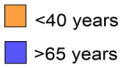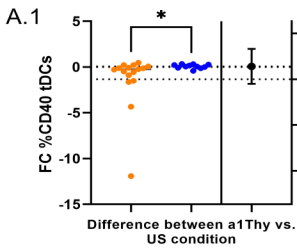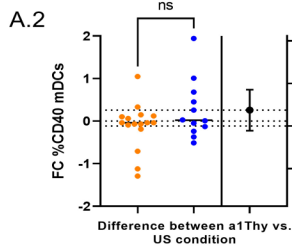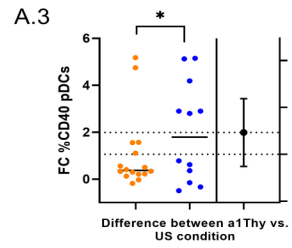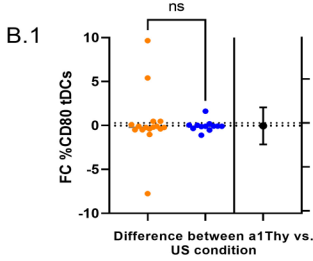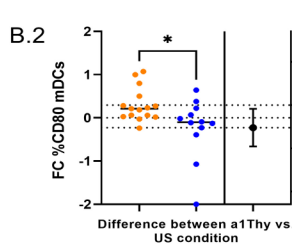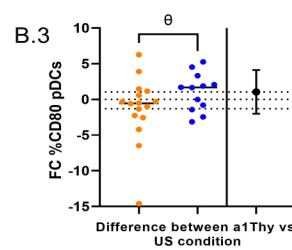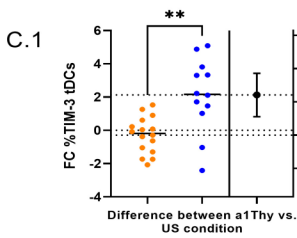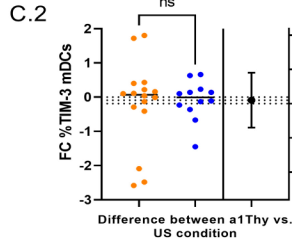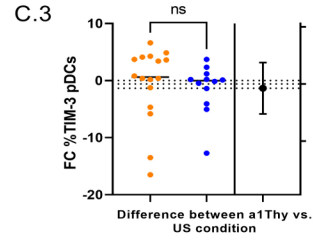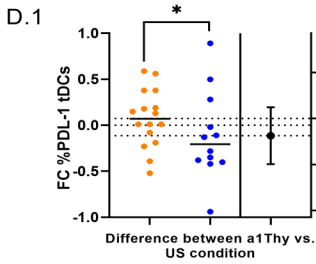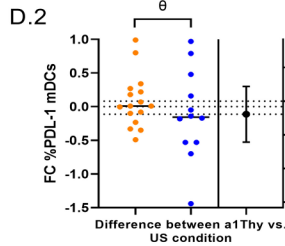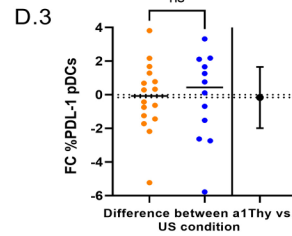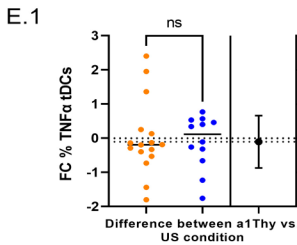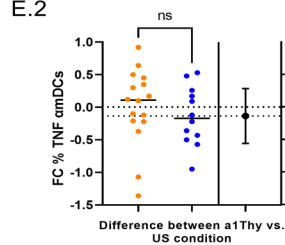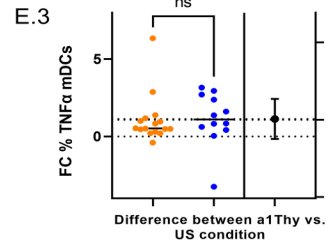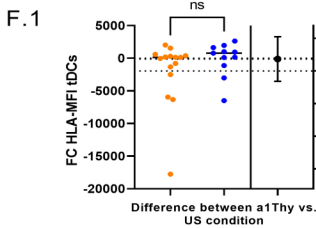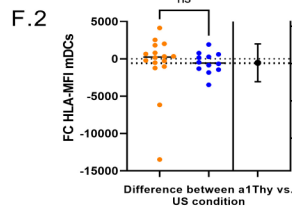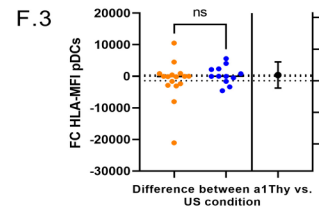

Supplement: Supplementary file 1 — Additional file 1. Fold change in DCs immunophenotyping. Scatter plot and fold change represents the difference between α1Thy treated condition and untreated condition in the marker expression on total DCS: CD40; CD80; TIM-3; PDL-1(A.1-D.1); intracellular production of TNFα(E.1) and intensity of fluorescence of HLADR(F:1). On mDCs: CD40; CD80; TIM-3; PDL-1(A.2-D.2); intracellular production of TNFα (E.2) and intensity of fluorescence of HLADR(F:2). On pDCs: CD40; CD80; TIM-3; PDL-1(A.3-D.3); intracellular production of TNFα(E.3) and intensity of fluorescence of HLADR(F.3). The medians with the interquartile ranges are shown. Ex-vivo: Ex-vivo condition; UT: Untreated condition; α1Thy: α1Thy treated condition. Each dot represents an individual. Orange dots represent < 40 years (n = 18) and > 65 years (n = 16) are highlighted with blue dots. U-Mann Whitney test was used comparing condition between different group (ns: no statistically significative, θ: p > 0.05 and < 0.1 *p < 0.05; **p < 0.01; ***p < 0.001). [file 12979_2023_351_MOESM1_ESM.pdf]

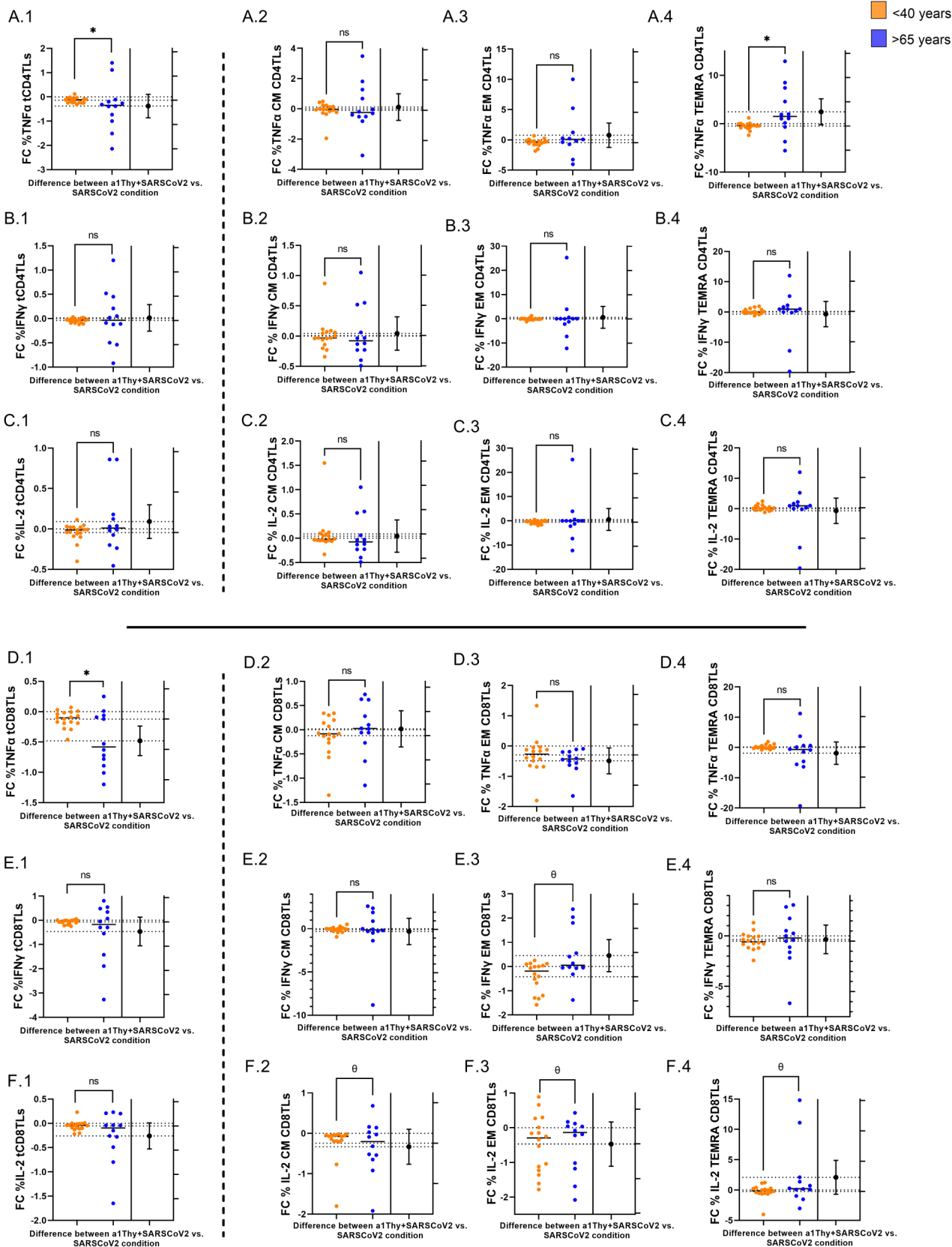

Supplement: Supplementary file 2 — Additional file 2. Fold change on total and memory CD4 + and CD8 + T lymphocytes. Scatter plot and fold change represents the difference between the SARS-CoV2 peptide-stimulated and α1Thy-pretreated condition and the SARS-CoV2 peptide-stimulated condition of cytokine production in total CD4 TLs: TNFα(A.1), IFNγ(B.1) and IL-2(C.1) and total CD8 TLs: TNFα(D.1), IFNγ(E.1) and IL-2(F.1); in memory CD4 TLs (CM,EM and TEMRA): TNFα(A.2-A.4); IFNγ(B.2-B.4); IL-2(C.2-C.4) and in memory CD8 TLs (CM,EM and TEMRA): TNFα(D.2-D.4); IFNγ(E.2-E.4); IL-2(F.2-F.4): The medians with the interquartile ranges are shown. Ex-vivo: Ex-vivo condition; UT: Untreated condition; α1Thy: α1Thy treated condition; SARS-CoV2: SARS-CoV2 peptides stimulation condition; α1Thy + SARS-CoV2: α1Thy treated and SARS-CoV2 peptides stimulation condition. Each dot represents an individual. Orange dots represent < 40 years (n = 18) and > 65 years (n = 16) are highlighted with blue dots. Memory populations represented in green (CM), in violet (EM) and in rose (TEMRA). Wilcoxon test was used comparing condition in the same group and U-Mann Whitney test was used comparing condition between different group (ns: not statistically significative, θ: p > 0.05 and < 0.1 *p < 0.05; **p < 0.01; ***p < 0.001). [file 12979_2023_351_MOESM2_ESM.pdf]

<40 years

3 cytokines response  
2 cytokines response  
1 cytokine response

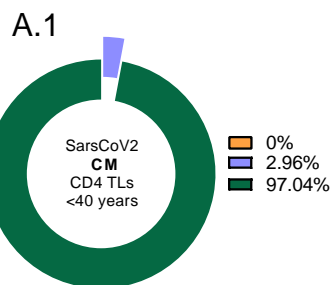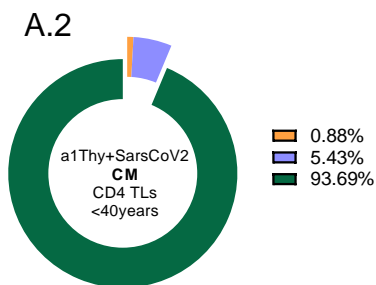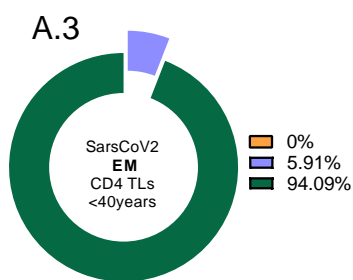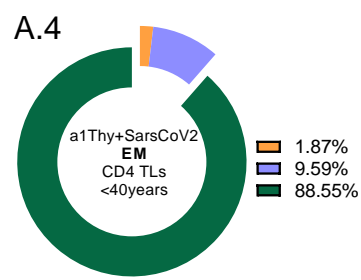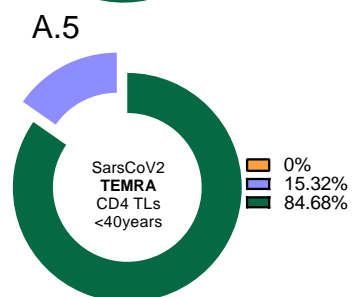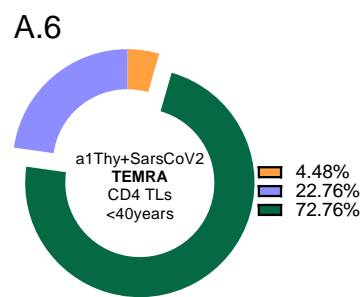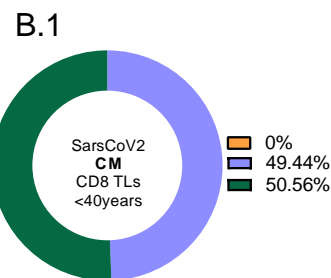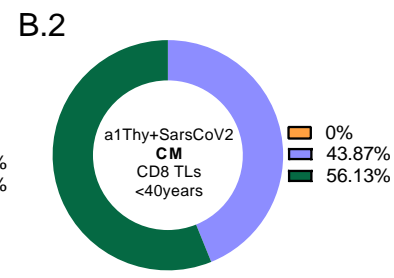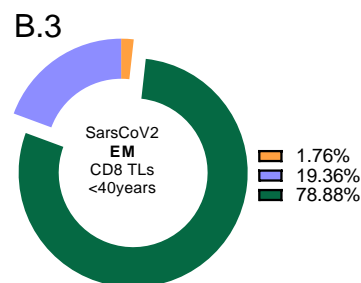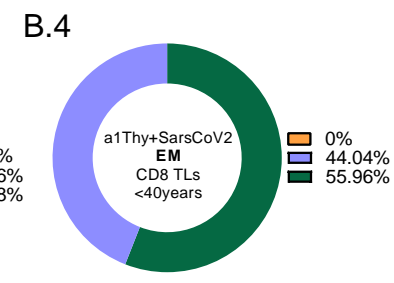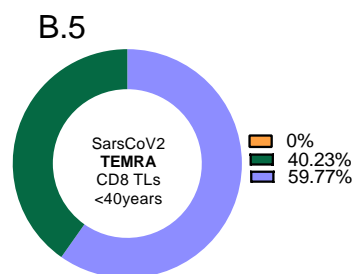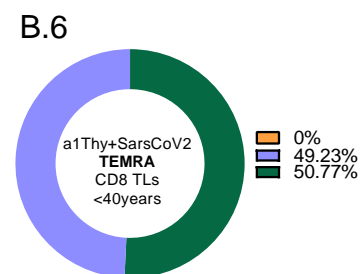

>65 years

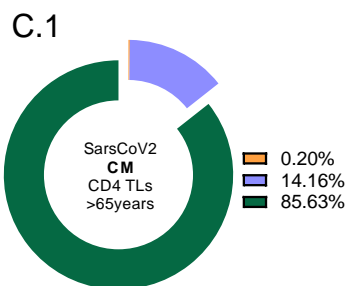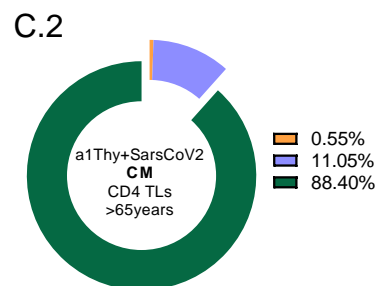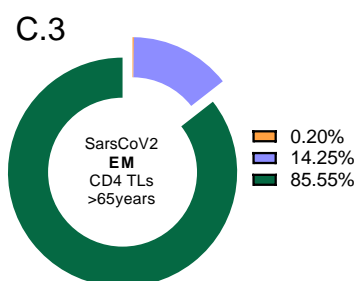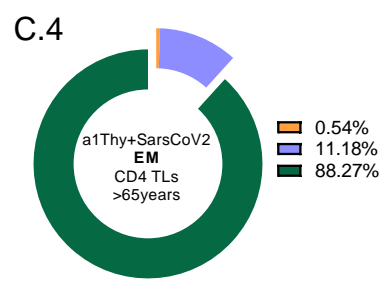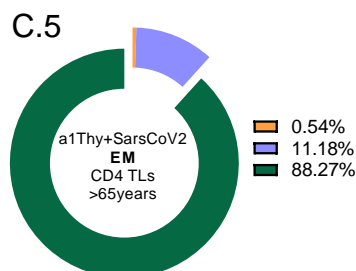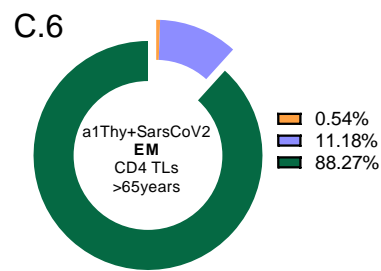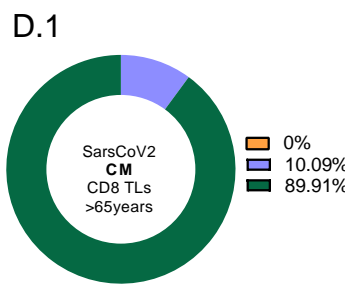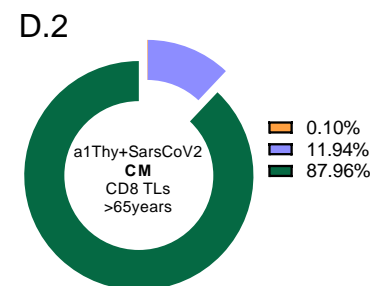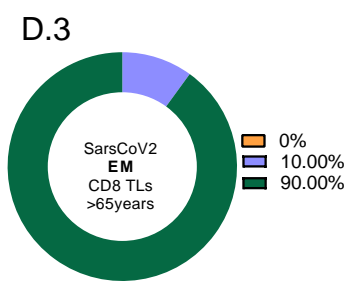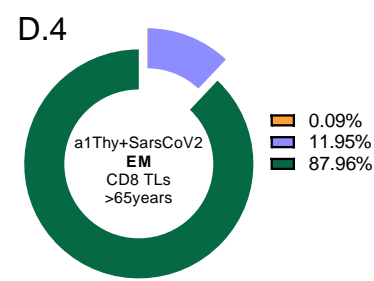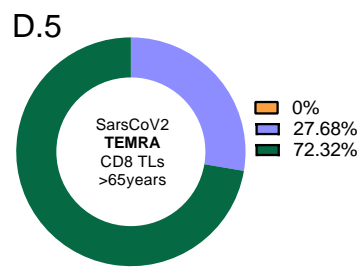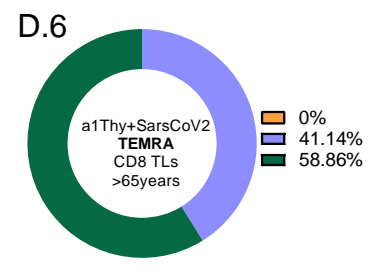

Supplement: Supplementary file 3 — Additional file 3. Polyfunctionality in memory CD4 + and CD8 + T cells. Polyfunctionality patterns of CD4 + and CD8 + memory T cells response to SARS-CoV2 producing one (green), two (blue) or three (orange) functions (combination of TNFα, IFNγ and IL-2) among the different condition (with or without α1Thy treatment) for < 40 years’ group in memory CD4 TLs (CM, EM and TEMRA) (A.1-A-A.6) and in memory CD8 TLs (CM, EM and TEMRA) (B.1-B.6) and for > 65 years in memory CD4 TLs (CM, EM and TEMRA) (C.1-A-C.6) and in memory CD8 TLs (CM, EM and TEMRA) (D.1-D.6). [file 12979_2023_351_MOESM3_ESM.pdf]

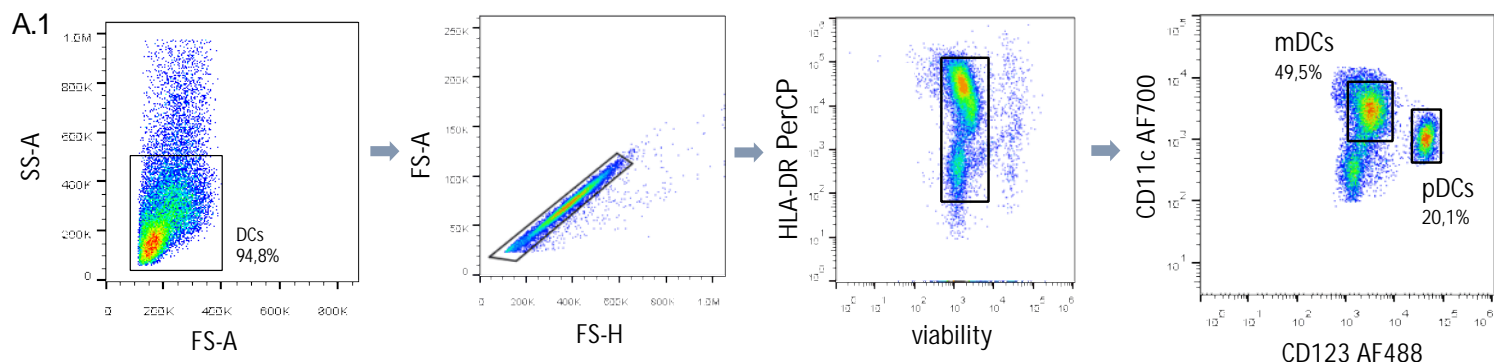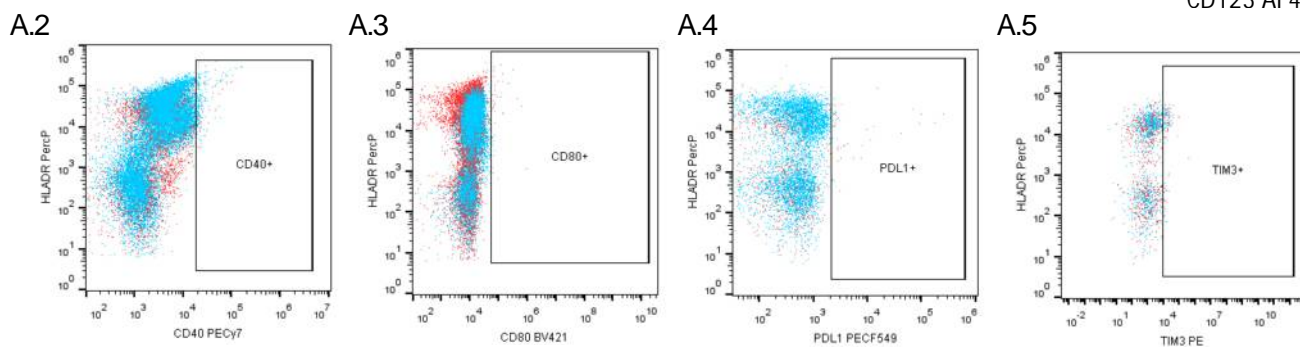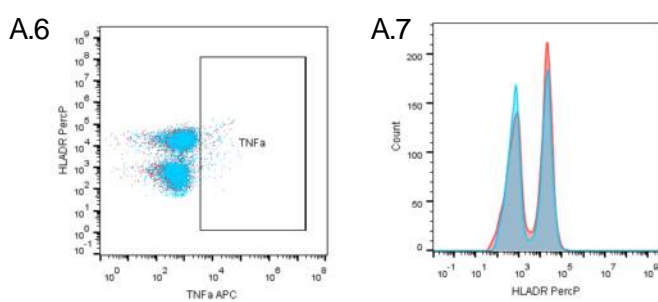

■ UT  
■ α1Thy

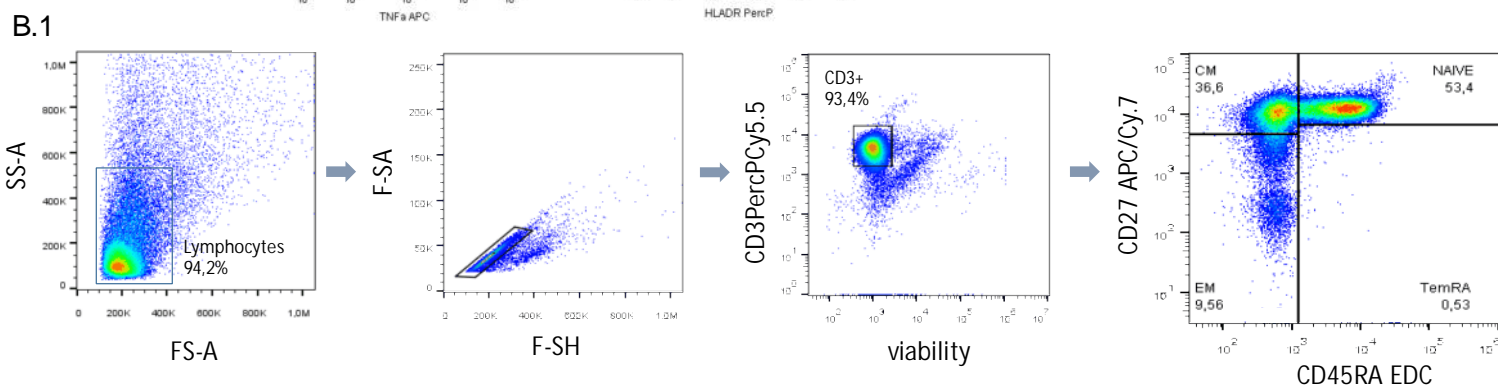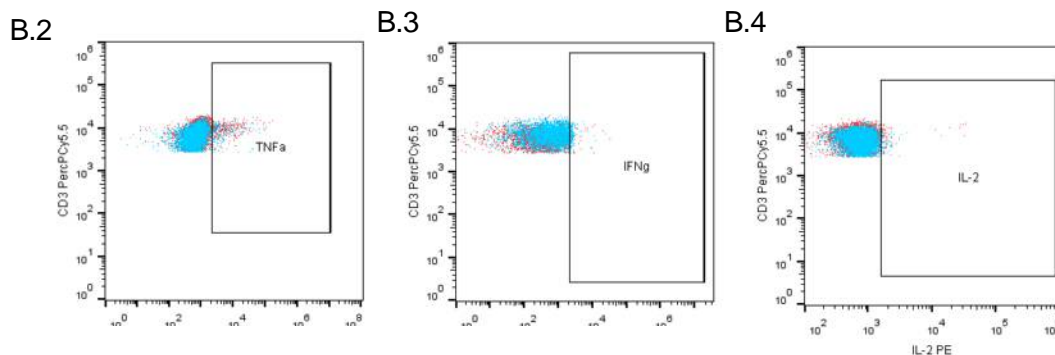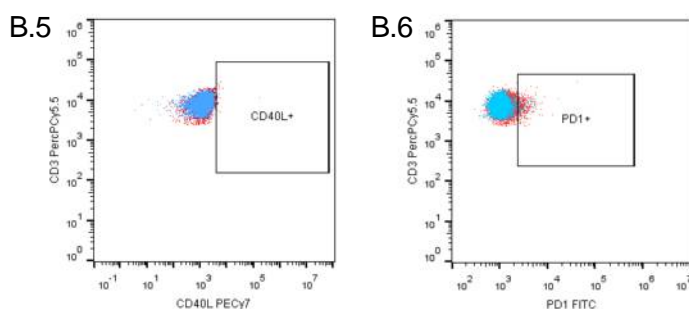

■ SARS-CoV2  
■ α1Thy+ SARS-CoV2

Supplement: Supplementary file 4 — Additional file 4. DCs and T-cells gating strategy from a donor. DCs gating strategy and pseudocolor plot representation of surface markers expression CD40, CD80, PDL1, TIM-3, TNFα production and HLADR intensity fluorescence histogram (A.1-A.7). T-cell gating strategy and pseudocolor plot representation of intracellular cytokine production TNFα, IFNγ, IL-2 and surface marker expression CD40L and PD1 (B.1-B.6). UT: untreated condition (red); α1Thy: α1Thy treated condition (blue); SARS-CoV2: SARS-CoV2 peptides stimulated condition (red); α1Thy + SARCoV2: α1Thy treated and SARS-CoV2 peptides stimulated condition (blue). [file 12979_2023_351_MOESM4_ESM.pdf]
